# Nutrition: a key environmental dietary factor in clinical severity and cardio-metabolic risk in psoriatic male patients evaluated by 7-day food-frequency questionnaire

**DOI:** 10.1186/s12967-015-0658-y

**Published:** 2015-09-16

**Authors:** Luigi Barrea, Paolo Emidio Macchia, Giovanni Tarantino, Carolina Di Somma, Elena Pane, Nicola Balato, Maddalena Napolitano, Annamaria Colao, Silvia Savastano

**Affiliations:** I.O.S. & COLEMAN Srl, Naples, Italy; Dipartimento di Medicina Clinica e Chirurgia, Medical School of Naples, Federico II University, Via Sergio Pansini, 5, 80131 Naples, Italy; Centro Ricerche Oncologiche di Mercogliano, Istituto Nazionale per lo studio e la Cura Dei Tumori “Fondazione Giovanni Pascale”, IRCCS, Mercogliano, Italy; IRCCS SDN, Napoli Via Gianturco 113, 80143 Naples, Italy

**Keywords:** Environmental dietary factor, 7-day food records, PASI, HoMA-IR, VAI, FLI, MUFA

## Abstract

**Background:**

Western dietary pattern is included among the environmental dietary factors involved in the pathogenesis of psoriasis. Nutritional data collection methods and gender differences might affect the association between diet and psoriasis. The 7-day food records is considered the “gold standard” of self-administered food frequency questionnaires. In this study, we evaluated the differences in the dietary intake, anthropometric measurements and cardio-metabolic risk profile in a group of psoriatic patients compared with an age and Body Mass Index (BMI)-matched control group. In addition, in the group of psoriatic patients we investigated the association between the dietary intake and clinical severity of psoriasis.

**Methods:**

Cross-sectional case control observational study. A total of 82 adult males, 41 treatment-naïve patients with psoriasis and 41 healthy subjects matched for age and BMI were included in the study. The clinical severity of psoriasis was by assessed by Psoriasis Area and Severity Index (PASI) score. The dietary interview data were collected by a 7-day food records. Anthropometric measures, glucose and lipid profile, liver function tests and C-reactive protein levels were measured. Homeostasis Model Assessment of Insulin Resistance (HoMA-IR), Visceral Adiposity Index (VAI) and the Fatty Liver Index (FLI) were calculated.

**Results:**

Psoriatic patients consumed a higher percentage of total and simple carbohydrates, total fat, polyunsaturated fatty acid (PUFA) and n-6/n-3 PUFAs ratio, and cholesterol, while the consumption of protein, complex carbohydrates, monounsaturated fatty acid (MUFA), n-3 PUFA and fiber was lower than in the control group. In addition, psoriatic patients presented altered anthropometric measurements, glucose and lipid profile, liver function tests, and elevated values of HoMA-IR, VAI and FLI. PASI score well correlated with anthropometric measures, glucose and lipid profile, liver function tests, cardio-metabolic indices, and the dietary components, except for protein and total carbohydrates. At logistic regression analysis between PASI score and MUFA, MetS presence was well predicted only by higher PASI score (OR = 1.794; p = 0.002; CI 1.242–2.591). At multiple regression analysis, MUFA was the best predictor of PASI score (*r*^*2*^ = 0.387, *β* = −0.635, *t* = −5.127, *p* < 0.001).

**Conclusion:**

Differences in dietary intake were observed in adult male psoriatic patients compared with the controls. These differences were associated to the severity of the psoriasis and cardio-metabolic risk. FLI represented an early indicator of the cardio-metabolic risk profile in psoriatic patients, and dietary MUFA were major predictor of the clinical severity of psoriasis, while the association between psoriasis and metabolic syndrome appeared to be independent of MUFA intake. The low MUFA consumption might act as a possible adjunctive mechanism in increasing the inflammation milieu of psoriatic patients.

## Background

Psoriasis is a common chronic inflammatory skin disease associated with obesity and serious comorbidities, such as type 2 diabetes mellitus, hypertension, metabolic syndrome [1], and hepatic steatosis (HS) [2], a major feature associated with the non-alcoholic fat liver disease (NAFLD) and the hepatic manifestation of metabolic syndrome [3]. It has been speculated that visceral obesity, insulin resistance and the release of inflammatory cytokines, may play a role in this association [4, 5].

The incidence of chronic inflammatory diseases is increased in Western countries over the past few decades [6]. In addition, males and females exhibit substantial differences in their eating behaviors in terms of energy intake, food consumption and nutrients intake [7, 8]. In particular, besides basic gender differences in weight concern and body self-perception, adults males consume more energy, saturated fat acid (SFA), and cholesterol but less complex carbohydrates and fibre than females. Specific macronutrient, such as simple carbohydrates, SFA and n-6 polyunsaturated fatty acid (n-6 PUFA) may contribute to a proinflammatory state, while others, including fiber, monounsaturated fat acid (MUFA), n-3 polyunsaturated fatty acid (n-3 PUFA), are associated with reduced levels of inflammatory [9].

The temporal sequence of the bidirectional relationships between inflammatory state induced by the Western dietary pattern, metabolic syndrome and psoriasis is still debated [10]. Indeed, the diet is included among risk factors for psoriasis [11] and dietary modifications have been reported to modulate the clinical course of the disease [12]. In particular, a reduced consumption of specific food items, such as black coffee, black tea, chocolate, yerba mate, pepper, smoked foods, beef and monosodium glutamate as flavor enhancer, and an increased consumption of foods rich in beta carotene have been strongly recommended, along with the suspension of alcoholic drinks and tobacco [13]. A protective effect of an energy-restricted diet enriched in n-3 PUFA on metabolic markers and clinical outcome in obese patients with psoriasis has been recently demonstrated [14]. Moreover, adherence to Mediterranean diet and in particular the consumption of some food items, such as extra-virgin olive oil (EVOO), the main source of MUFA, have been linked to the severity of the disease in a group of psoriatic patients of both gender [15].

Observational studies describing the association between diet and psoriasis have been commonly performed including psoriatic patients of both gender and using self-report methods. To date, a careful evaluation of dietary intake using the 7-day food records, considered the “gold standard” of self-administered food frequency questionnaires [16, 17], has not been yet provided, and no studies evaluating separately the dietary intake in males and females with psoriasis are available. Moreover, the large majority of studies have included patients in treatment with anti-psoriatic agents, such as biologics, systemic corticosteroids or methotrexate, which may have interfered with anthropometric measures and blood glucose and lipid profile [18]. Finally, in the setting of the inflammation-related insulin resistance, the association between psoriasis and other cardio-metabolic risk indices, such as the Visceral Adiposity Index (VAI), a gender-specific indicator of adipose distribution and dysfunction [19], and the Fatty Liver Index (FLI), as an accurate predictor of HS [20] has not yet evaluated.

Aim of the present study is to compare the dietary intake (using a 7-day food records), anthropometric measurements and cardio-metabolic risk profile (defined by the Homeostasis Model Assessment of Insulin Resistance (HoMA-IR), VAI and FLI) between male psoriatic patients and healthy control subjects matched by age and Body Mass Index (BMI). In addition, in the group of psoriatic patients we investigated the association between the dietary intake and clinical severity of psoriasis to evidence a possible involvement of the diet-induced proinflammatory state as adjunctive mechanism increasing the inflammation milieu of psoriatic patients.

## Methods

### Design and setting

This is a cross-sectional case–control observational study carried out at the Department of Clinical Medicine and Surgery of the University of Naples Federico II (Italy). The work has been carried out in accordance with the Code of Ethics of the World Medical Association (Declaration of Helsinki) for experiments involving humans, and it has been approved by the Ethical Committee of the University of Naples “Federico II” Medical School. The purpose of the protocol was explained to both the patients and the controls, and written informed consent was obtained.

The study was conducted without support from the pharmaceutical industry.

### Population study

The study has been conducted on 41 adult treatment-naïve patients out of 254 unselected Caucasian subjects of both gender affected by psoriasis attending the Psoriasis Care Center of the Outpatient Clinic of the Section of Dermatology, University of Naples Federico II from July 2013 to July 2014. In order to improve the power of the study, we increased the homogeneity of the patient sample by including treatment-naïve adult male patients only. Patients were excluded from the study if they (1) were females (122 patients); (2) had a diagnosis of psoriasis lasting >6 months or were receiving any systemic treatment for psoriasis including acitretin, ciclosporin, methotrexate, phototherapy or biologics for at least 3 months (48 patients); (3) had a diagnosis of pustular (2 patients), erythrodermic (1 patient) or arthropathic psoriasis (13 patients); (4) had received any drug therapy known to affect carbohydrate or lipid metabolism during the previous 6 months (6 patients); (5) had a history of excessive alcohol use (2 patients); (6) were current smokers (15 patients); (7) neoplastic, metabolic, hepatic, and cardiovascular disorder or other concurrent medical illness (i.e. renal disease, and malabsorptive disorders) (4 patients).

Forty-one healthy subjects were chosen as controls among hospital volunteers and employees from the same geographical area. Controls were matched by age and BMI and had comparable education, employment, marital status, or participation in physical activity as the study group. Exercise pattern and type were noted by asking orally.

The Psoriasis Area and Severity Index (PASI) score is a validated and widely used tool for measuring psoriasis severity [21]. The scale evaluates four areas of the body (head/neck, upper limbs, trunk, and lower limbs) for erythema, scaliness and thickness of psoriatic plaques. The PASI score can range from 0 to 72, with higher scores indicating greater severity. To prevent rate biases the dermatologists who evaluated the PASI score were blinded to the design of the study.

### Dietary assessment

These data were carried out during a face-to-face interview between the patient and a certified nutritionist. The dietary interview allowed to quantify foods and drinks by using a photographic food atlas (≈1000 photographs) of known portion sizes to ensure accurate completion of the records. Moreover, dietary data, including beverage intakes and alcohol consumption, were collected by a 7-day food records. The subjects returned the records to the nutritionist who asked supplemental questions if necessary. Data were stored and processed using a commercial software (Terapia Alimentare Dietosystem^®^ DS-Medica, http://www.dsmedica.info). Considering the quantities and qualities of foods consumed, this database, that included about 300 food items, was able to calculate not only the daily caloric intake but also the quantities of macronutrients: protein; total, complex and simple carbohydrates; total fat, SFA, MUFA, PUFA (n-6 PUFA, n-3 PUFA and n-6/n-3 PUFAs ratio); cholesterol and fibers. The dietary macronutrient composition was drawn by the tables of food composition and recommended dietary intakes of the BDA (Food Composition Database for Epidemiological Studies in Italy, European Institute of Oncology, http://www.bda-ieo.it/).

### Anthropometric measurements

All anthropometric measurements were taken with subjects wearing only light clothes and without shoes. In each subject, weight and height were measured to calculate the BMI [weight (kg) divided by height squared (m^2^), kg/m^2^]. Height was measured to the nearest 1 cm using a wall-mounted stadiometer. Body weight was determined to the nearest 50 g using a calibrated balance beam scale. Waist circumference (WC) was measured to the closest 0.1 cm using a non-stretchable measuring tape at the natural indentation or at a midway level between lower edge of the rib cage and iliac crest if no natural indentation was visible. The measurement was made with the subject standing upright, feet together and arms hanging freely at the sides, without compression of the soft tissue. According to the National Cholesterol Education Program’s Adult Treatment Panel III (NCEP-ATP III) criteria, abdominal obesity was defined as WC ≥102 cm.

### Biochemical measurements

Samples were collected in study population between 8 and 10 a.m. after an overnight fast of at least 8 h and stored at −80 °C until processed. All biochemical analyses including fasting plasma glucose, total cholesterol, triglycerides, aspartate aminotransferase (AST), alanine transaminase (ALT), γ-glutamyltransferase (γGT) were performed with a Roche Modular Analytics System in the Central Biochemistry Laboratory of our Institution. Low-density lipoprotein (LDL) and high-density lipoprotein (HDL) cholesterol were determined by direct method (homogeneous enzymatic assay for the direct quantitative determination of LDL and HDL cholesterol). C-Reactive Protein (CRP) levels were determined with a nephelometric assay with CardioPhase high-sensitive from Siemens Healthcare Diagnostics (Marburg, Germany). Fasting insulin levels were measured by a solid-phase chemiluminescent enzyme immunoassay using a commercially available kits (Immunolite Diagnostic Products Co, Los Angeles, CA). The intra-assay coefficients of variations (CV) were <4 % for CRP and <5.5 % for insulin. HoMA-IR was calculated according to Matthews et al. [22]: a value of HoMA-IR >2.0 was set as stringent measure of insulin resistance. VAI score was calculated using the following gender-specific formula, males [23]: [(WC/39.68) + (1.88 × BMI) × (triglycerides mg/dl × 0.0113/1.03) × (1.31/HDL cholesterol mg/dl × 0.0259)]. The median 4.9 value was used as cut-off value [24]. FLI was calculated with the formula: [FLI = e^L^/(1 + e^L^) × 100, L = 0.953 × log_e_ triglycerides + 0.139 BMI + 0.718 × log_e_γGT + 0.053 × WC − 15.745]. FLI of 30 was considered as the cut-off value on the basis of Bedogni’s criterion [20].

### Criteria to define metabolic syndrome

According to the NCEP ATP III definition, metabolic syndrome (MetS) is present if three or more of the following five criteria are met: waist circumference over 102 cm (men), blood pressure over 130/85 mmHg, fasting triglyceride level over 150 mg/dl, fasting high-density lipoprotein cholesterol level less than 40 mg/dl (men) and fasting glucose over 100 mg/dl [25]. Blood pressure was measured using a standard sphygmomanometer in the supine position, after a rest of at least 5 min.

### Ultrasound evaluation

The presence of HS, commonly known as “bright liver”, was assessed with a US diagnostic system (Logiq P5, General Electric, Milan, Italy) with a 3.5-MHz convex probe. All the US determinations were made by the same trained operator. The intra-operator variability, as evaluated in 20 subjects within 1 week from the first ultrasonographic examination, showed an overall r value of 0.92 (p < 0.001). As previously validated [26], the presence/severity of HS was defined by comparing the echogenicity of the liver and of the kidney cortex: Grade 0 (absent): iso-echogenicity; Grade 1 (mild): diffuse and homogeneous hyper-echogenicity; Grade 2 (moderate): attenuation of the ultrasonic beam rear; Grade 3 (severe): lack of diaphragm profile visualization. Technically, echo intensity can be influenced by many factors, particularly gain intensity. To avoid confounding factors that could modify echo intensity and thus bias comparisons, mean brightness levels of both liver and right kidney cortex were obtained on the same longitudinal sonographic plane.

### Statistical analysis

Results are expressed as mean ± SD or as median plus range according to variable distributions evaluated by Kolmogorov–Smirnov test. To correct for skewed distributions, CRP levels and HoMA-IR values were logarithmically transformed and back-transformed for presentation in tables. Differences between groups were analyzed by paired *t* test or Wilcoxon signed-rank test, when appropriate. The chi 2 (*χ*^2^) test was used to test the significance of differences in frequency distribution. In the group of psoriatic patients, the correlations between variables were performed using Pearson *r* or Spearman’s *rho* correlation coefficients. The presence of significant associations of the presence of MetS, as dependent variable, with PASI score and MUFA in the psoriatic group was analyzed using logistic regression, and odds ratio (OR) and 95 % confidence interval (CI) were computed.

In addition, in the group of psoriatic patients, three multiple linear regression analysis models (stepwise method), expressed as *r*^*2*^, Beta (*β*) and *t*, with PASI score as dependent variables were used to estimate the predictive value of: (1) HoMA-IR, VAI and FLI; (2) complex and simple carbohydrates, total fat, MUFA and PUFA (n-6 PUFA, n-3 PUFA and n-6/n-3 PUFAs ratio); (3) FLI and MUFA. In these analyses, we entered only those variables that had a *p* value <0.05 in the univariate analysis (partial correlation). To avoid multicollinearity, variables with a variance inflation factor (VIP) >10 were excluded. Values ≤5 % were considered statistically significant. The power sample was calculated by the differences of means + SD of MUFA in each group. Data were stored and analyzed using the MedCalc^®^ package (Version 12.3.0 1993–2012 MedCalc Software bvba—MedCalc Software, Mariakerke, Belgium).

## Results

With a type I (alpha) error, of 0.05 (95 %), and with a type II (beta) of 0.10, (90 %), the number of cases required for each group was set at 43, near to the ours. Socio-demographic, anthropometric and metabolic characteristics of the treatment-naïve patients with psoriasis and the subjects matched for age and BMI serving as control group are shown in Table 1. Socio-demographic characteristics and participation in physical activity were not significantly different in both groups, while the metabolic risk factors and liver function tests were significantly higher in psoriatic patients than in control group. In particular, logarithmically transformed CRP levels and HoMA-IR in psoriatic patients were significantly higher than in controls [CRP: 0.1 (−2.3 − 3.4) *vs* −0.2 (−2.3 − 0.7), respectively; p = 0.050; HoMA-IR: 1.0 (−1.2 − 2.7) vs −0.1 (−3.2 − 2.9), respectively; p = 0.001]. CRP levels and HoMA-IR values have been back-transformed for presentation in Table 1. HS was diagnosed in 95.1 % psoriatic patients (39 patients) vs 48.7 % controls (20 subjects), *χ*^2^ = 19.578, *p* < 0.001; in particular, grade 2 and 3 HS was present in 25 psoriatic patients (60.8 %) vs 10 controls (24.4 %), *χ*^2^ = 9.770, *p* = 0.002. In addition, HoMA-IR, VAI and FLI were also significantly higher in psoriatic patients. HoMA-IR values ≥2 occurred significantly more frequently among case-patients than controls: 68.3 % (28 patients) vs 26.8 % (11 controls), *χ*^2^ = 14.173, *p* < 0.001. Similarly, VAI and FLI >cut-off values occurred significantly more frequently among psoriatic patients than controls: VAI: 95.1 % (39 patients) vs 75.6 % (31 controls), *χ*^2^ = 4.783, *p* = 0.029; FLI: 78.0 % (32 patients) vs 51.2 % (21 controls), *χ*^2^ = 5.335, *p* = 0.021. Table 2 shows the prevalence of the metabolic risk factors in the two groups. Although systolic/diastolic blood pressure values and fasting glucose levels were found to be higher among case-patients than controls, the prevalence of MetS was not statistically different in the two groups.

**Table 1:** Socio-demographic, anthropometric and metabolic characteristics of psoriatic patients and control group

Parameters	Psoriatic patients n = 41	Control group n = 41	*p* values
Age (years)	52.1 ± 11.1	49.7 ± 10.0	0.309
BMI (kg/m^2^)	31.2 ± 4.4	30.1 ± 6.9	0.407
Education status			*χ* ^2^ = 0.049 p = 0.825
Graduates (n)	20	22	
Higher secondary (n)	21	19	
Employment			*χ* ^2^ = 1.384 p = 0.239
Un-employed (n)	3	0	
Employed (n)	38	41	
Marital status			*χ* ^2^ = 0.139 p = 0.710
Single (n)	5	3	
Married (n)	36	38	
Physical activity			*χ* ^2^ = 0.625 p = 0.429
Sedentary (n)	39	36	
Moderate (n)	2	5	
SBP (mmHg)	126.7 ± 15.0	116.6 ± 11.8	*0.002*
DBP (mmHg)	80.0 ± 9.7	71.3 ± 5.7	*<0.001*
WC (cm)	108.1 ± 13.8	102.6 ± 8.4	*0.035*
CRP levels (ng/ml)*	3.7 ± 6.4	0.8 ± 0.5	*0.050*
Fasting Glucose (mg/dl)	111.7 ± 34.2	87.9 ± 32.8	*0.004*
Insulin (μU/ml)	11.1 ± 6.2	7.4 ± 7.6	*0.022*
Total cholesterol (mg/dl)	219.0 ± 44.5	199.2 ± 47.9	*0.021*
HDL cholesterol (mg/dl)	38.9 ± 11.8	45.3 ± 11.9	*0.025*
LDL cholesterol (mg/dl)	147.3 ± 49.0	124.4 ± 49.2	*0.035*
Triglycerides (mg/ml)	164.4 ± 93.5	127.4 ± 48.8	*0.041*
AST (U/l)	32.4 ± 15.5	31.9 ± 22.9	0.932
ALT (U/l)	37.3 ± 17.0	26.3 ± 12.0	*0.007*
γGT (U/l)	43.8 ± 23.8	31.3 ± 20.9	*0.030*
HoMA-IR*	3.4 ± 2.9	2.0 ± 3.3	*0.001*
VAI	5.4 (1.3–23.6)	4.1 (1.1–8.6)	*0.049*
FLI	76.1 ± 23.6	60.4 ± 26.9	*0.009*

The psoriatic patients exhibited statistically significant differences compared with controls for anthropometric measurements, metabolic profiles and cardio-metabolic indices. Results are expressed as mean ± SD or as median plus range according to variable distributions evaluated by Kolmogorov–Smirnov test. *CRP levels and *HoMA-IR values were logarithmically transformed and back-transformed for presentation in table. Differences between groups were analyzed by paired t test or Wilcoxon signed-rank test, when appropriate. The Chi-square (*χ* ^2^) test was used to test the significance of differences between the two groups. Italicized p-values represent significant differences at p < 0.05

*BMI* Body Mass Index, *SBP* systolic blood pressure, *DBP* diastolic blood pressure, *WC* Waist Circumference, *CRP* C-reactive protein, *HDL* High-density lipoprotein, *LDL* low-density lipoprotein, *AST* aspartate aminotransferase, *ALT* Alanine transaminase, *γGT* γ-glutamyltransferase, *HoMA*-*IR* Homeostatic Model Assessment for Insulin Resistance, *VAI* Visceral Adiposity Index, *FLI* Fatty Liver Index, *MetS* Metabolic Syndrome

**Table 2:** Frequency of metabolic risk factors in psoriatic patients and control group

Parameters	Psoriatic patients n = 41		Control group n = 41		*χ* ^2^	*p* values
	n	%	n	%		
WC (≥102 cm)	27	65.9	21	51.2	1.26	0.262
SBP/DBP (≥130/85 mmHg)	17	41.5	7	17.1	4.77	*0.029*
Triglycerides (≥150 mg/dl)	17	41.5	16	39.0	0.00	1.000
HDL cholesterol (≤40 mg/dl)	16	39.0	10	24.4	1.41	0.235
Fasting glucose (≥100 mg/dl)	26	63.4	13	31.7	7.04	*0.008*
*MetS diagnosis*	17	41.1	9	22.0	2.76	0.097

The psoriatic patients exhibited statistically significant differences compared with controls for systolic and diastolic blood pressure and fasting glucose. According to the NCEP ATP III definition, the metabolic syndrome is defined as the coexistence of three or more of the following findings: (1) Increased waist circumference. (2) Hypertriglyceridaemia. (3) Hypertension. (4) Elevated fasting plasma glucose. (5) Low high-density lipoprotein (HDL) cholesterol level. Results are expressed as number and percentage. The Chi square (*χ* ^2^) test was used to test the significance of differences between the two groups. Italicized p-values represent significant differences at p < 0.05

*WC* Waist Circumference, *SBP* systolic blood pressure, *DBP* diastolic blood pressure, *HDL* High-density lipoprotein, *MetS* Metabolic Syndrome

**Fig. 1:**
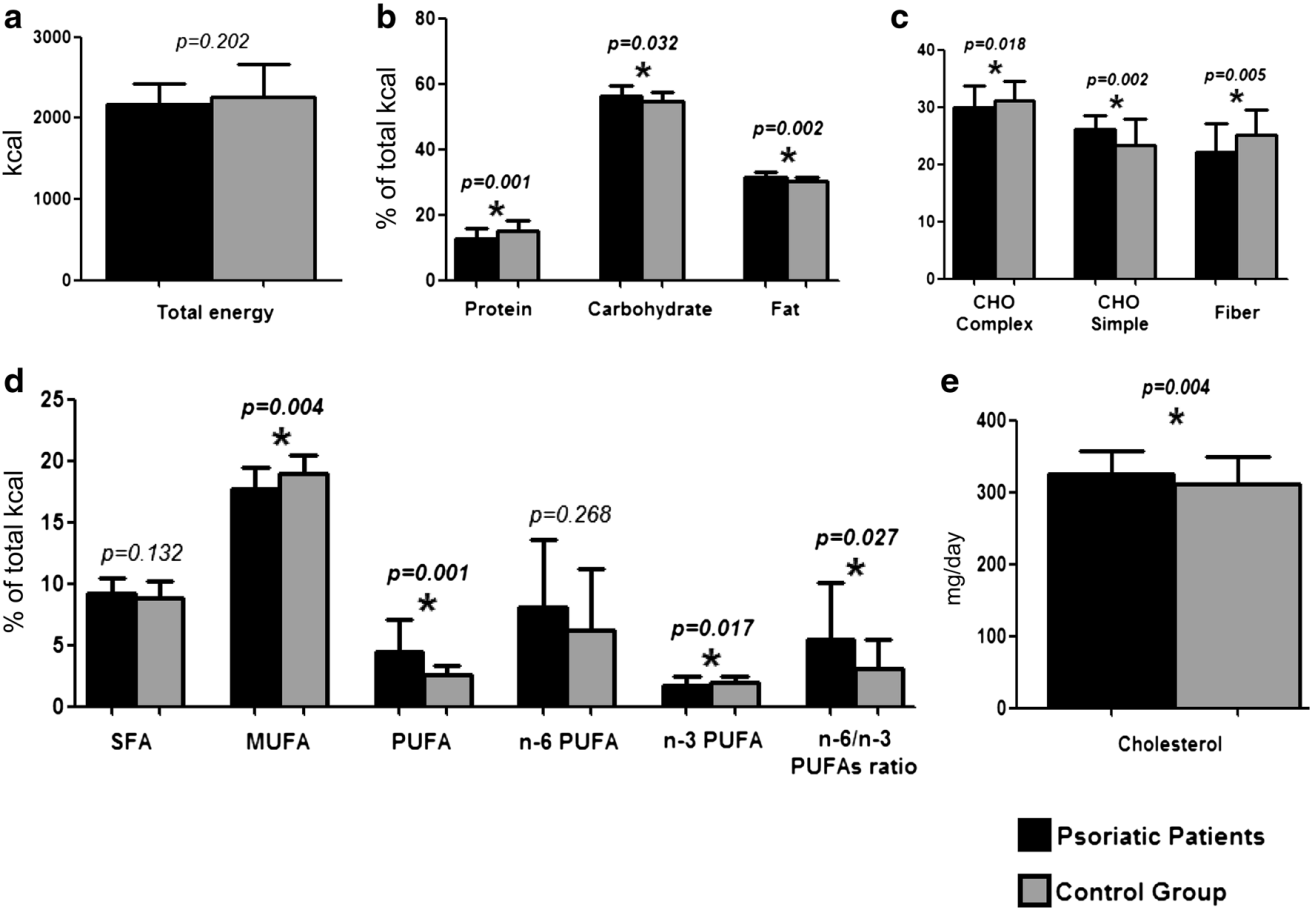
Dietary pattern in psoriatic patients and control group. In the figure are shown the dietary differences between psoriatic patients and control subjects. **a** Total calories (kcal). **b** Percentage of macronutrients in diet. Panel C Carbohydrates (CHO) (% of total charbohydrates) and fibers (g/day). **d** Fats (*SFA* saturated fatty acids; *MUFA* monounsaturated fatty acids; *PUFA* polyunsaturated fatty acids.). **e** Cholesterol. Psoriatic patients have a higher consumption of total and simple carbohydrates, total fat, PUFA, n-6/n-3 PUFAs ratio, and cholesterol, while the consumption of protein, complex carbohydrates, MUFA, n-3 PUFA and fiber, is lower than in the control group. No statistically significant differences of a total energy, SFA and n-6 PUFA were observed between the two groups

**Fig. 2:**
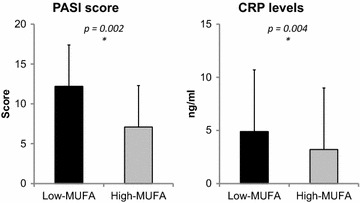
PASI score and CRP levels in psoriatic patients on the basis of MUFA intake. Psoriatic patients were divided in two groups on the basis of median percentage of MUFA intake (18.2 %). Patients with MUFA intake below median (Low-MUFA) presented higher values of PASI score and CRP levels when compared with patients having a MUFA intake higher than the median (High-MUFA).* PASI* Psoriasis Area and Severity Index, * CRP* C-reactive protein

The total energy and nutrient intake in psoriatic patients and control group are reported in Fig. 1. All participants to the study completed the 7-day food records. The dietary survey revealed that the psoriatic patients have a higher consumption of total and simple carbohydrates, total fat, PUFA, n-6/n-3 PUFAs ratio, and cholesterol, while the consumption of protein, complex carbohydrates, MUFA, n-3 PUFA and fiber, is lower than in the control group. No statistically significant differences of a total energy, SFA and n-6 PUFA were observed between the two groups. However, when the psoriatic patients were divided in two groups based on median percentage of MUFA intake (18.2 %), patients with low MUFA intake presented higher values of PASI score and CRP levels than their pairs with high MUFA intake (Fig. 2).

### Correlation studies

Among psoriatic patients the mean value of PASI score was 9.0 ± 7.0 (range 0.1–28.3). Besides the negative correlation with HDL-cholesterol, PASI score directly correlated with anthropometric, metabolic and cardio-metabolic parameters (Table 3). After adjusting for BMI, all the associations between PASI score and the study variables were preserved, except for AST. At multiple regression analysis, among HoMA-IR, VAI and FLI, the latter was the major predictor of PASI score (*r*^*2*^ = 0.358, *β* = 0.611, *t* = 4.823, *p* < 0.001).

**Table 3:** Correlations among PASI score and study variable in psoriatic patients

Simple correlation			Partial correlation (adjusting for BMI)	
Parameters PASI score		*p* values	PASI score	*p* values
Age (years)	−0.151	0.347	−0.095	0.560
BMI (kg/m^2^)	0.532	*<0.001*	–	–
SBP (mmHg)	0.883	*<0.001*	0.906	*<0.001*
DBP (mmHg)	0.716	*<0.001*	0.808	*<0.001*
WC (cm)	0.736	*<0.001*	0.613	*<0.001*
CRP levels (ng/ml)	0.427	*0.005*	0.345	*0.029*
Fasting Glucose (mg/dl)	0.471	*0.002*	0.502	*0.001*
Insulin (μU/ml)	0.409	*0.008*	0.466	*0.002*
Total cholesterol (mg/dl)	−0.123	0.444	−0.133	0.412
HDL cholesterol (mg/dl)	−0.528	*<0.001*	−0.514	*0.001*
LDL cholesterol (mg/dl)	−0.145	0.367	−0.136	0.402
Triglycerides (mg/ml)	0.420	*0.006*	0.367	*0.020*
AST (U/l)	0.313	*0.046*	0.223	0.167
ALT (U/l)	0.498	*0.001*	0.411	*0.009*
γGT (U/l)	0.516	*0.001*	0.476	*0.002*
HoMA-IR	0.485	*0.001*	0.492	*0.001*
VAI	0.580	*<0.001*	0.544	*<0.001*
FLI	0.611	*<0.001*	0.373	*0.018*
HS	0.388	*0.012*	0.311	*0.050*
MetS (n. of parameters)	0.758	*<0.001*	0.683	*<0.001*

At simple correlation, PASI score was significantly association with BMI, WC, CRP levels, metabolic profile, liver function tests, and the cardio-metabolic indices. After adjusting for BMI the associations between PASI score and the study variables were mainly preserved. Correlations between variables were performed using Pearson *r* and Spearman’s *rho* correlation coefficients. Italicized p-values represent significant differences at p < 0.05

*BMI* Body Mass Index, *SBP* systolic blood pressure, *DBP* diastolic blood pressure, *WC* Waist Circumference, *CRP* C-reactive protein, *HDL* High-density lipoprotein, *LDL* low-density lipoprotein, *AST* aspartate aminotransferase, *ALT* Alanine transaminase, *γGT* γ-glutamyltransferase, *HoMA*-*IR* Homeostatic Model Assessment for Insulin Resistance, *VAI* Visceral Adiposity Index, *FLI* Fatty Liver Index, *HS* Hepatic Steatosis, *MetS* Metabolic Syndrome

In Table 4 is reported the correlation between PASI score, total energy and nutrient intake. PASI score correlated with all the dietary macronutrients, except for protein and total carbohydrates. After adjusting for BMI and number of parameters of Mets, the associations between PASI score and nutrient intake were mainly preserved. At logistic regression analysis between PASI score and MUFA, the MetS presence was well predicted only by higher PASI score (OR = 1.794; p = 0.002; CI 1.242–2.591). At multiple regression analysis among macronutrients, MUFA remained the major predictor of PASI score (*r*^*2*^ = 0.387, *β* = −0.635, *t* = −5.127, *p* < 0.001). Finally, at multiple regression analysis, PASI score was better predicted by MUFA than FLI (*r*^*2*^ = 0.387, *β* = −0.635, *t* = −5.127, *p* < 0.001 vs *r*^*2*^ = 0.532, *β* = 0.426, *t* = 3.619, *p* = 0.001).

**Table 4:** Correlation between PASI score with energy and nutrient intake

Simple correlation			Partial correlation (adjusting for BMI and metabolic syndrome)	
Parameters	PASI score		PASI score	
	*r*	*p* values	*r*	*p* values
Total energy (kcal)	0.432	*0.005*	0.022	0.895
Protein (% of total kcal)	−0.083	0.604	0.006	0.972
Carbohydrate (% of total kcal)	−0.164	0.305	−0.188	0.251
Complex (% of total kcal)	−0.471	*0.002*	−0.357	*0.026*
Simple (% of total kcal)	0.564	*<0.001*	0.352	*0.028*
Fat (% of total kcal)	0.491	*0.001*	0.372	*0.020*
SFA (% of total kcal)	0.413	*0.007*	0.289	0.075
MUFA (% of total kcal)	−0.635	*<0.001*	−0.460	*0.003*
PUFA (% of total kcal)	0.523	*<0.001*	0.384	*0.016*
n-6 PUFA (g/day)	0.514	*0.001*	0.321	*0.047*
n-3 PUFA (g/day)	−0.505	*0.001*	−0.547	*<0.001*
n-6/n-3 PUFAs ratio	0.621	*<0.001*	0.532	*<0.001*
Cholesterol (mg/day)	0.443	*0.004*	0.112	0.498
Fiber (g/day)	−0.471	*0.002*	−0.115	0.487

PASI score well correlated with all the dietary components, except for protein and total carbohydrates. Correlations between variables were performed using Pearson *r* and Spearman’s *rho* correlation coefficients. After adjusting for BMI and number of parameters of metabolic syndrome, the associations between PASI score and nutrient intake were mainly preserved. Italicized p-values represent significant differences at p < 0.05

*SFA* saturated fatty acids, *MUFA* monounsaturated fatty acids, *PUFA* polyunsaturated fatty acids

## Discussion

The main result of this study indicates that psoriatic patients, compared to the control group, have a higher consumption of simple carbohydrates, total fat and n-6/n-3 PUFAs ratio, with a lower intake of protein, complex carbohydrates, MUFA, n-3 PUFA and fiber. In addition, psoriatic patients have elevated HoMA-IR, VAI and higher prevalence both of MetS and HS, as evidenced by liver ultrasound and higher FLI values. These findings are in line with the current literature on the association between psoriasis and fatty liver in non-alcoholic fatty liver disease (NAFLD) and could be explained by the inflammatory burden of psoriasis acting on fatty liver development also independently of the metabolic derangements commonly reported among psoriatic patients [27, 28].

In the group of psoriatic patients we have further investigated the association between nutrition and psoriasis by correlating dietary macronutrient intake, clinical severity of psoriasis and altered cardio-metabolic profile. In particular, also adjusting for BMI and MetS, the correlations between PASI score and nutrient intake were mainly preserved. Our study is the first evidence reporting that in psoriatic patients the high consumption of simple carbohydrates is associated with the clinical severity of the disease.

Single food components have been suggested to play a role in etiology and pathogenesis of psoriasis [29]. Previous studies on the relationship of diet and nutrition with psoriasis have focused on either individual nutrients (e.g. fish oil, n-3 PUFA, vitamin B_12_, vitamin D, vitamin A, selenium, inositol and zinc and antioxidants) or individual food groups (e.g. fruit, vegetables, and fish [30]. However, diet is a complex combination of foods from various groups and nutrients, and some nutrients are highly correlated, thus it is very challenging, in free-living populations, to separate the effect of a single nutrient or food group from others [31]. In addition, it is well known that gender, age and educational status influence food choice behaviors [7]. In particular, women’s dietary profiles is characterized by a higher carbohydrates intake, including fruit and vegetables [8]. In the extensive survey on dietary intake and nutritional status in psoriatic patients of both gender using the 2003–2006 NHANES data [11], it has been shown an association between psoriasis and lower intake of simple carbohydrates. This association was largely unexpected and in contrast with literature’s data indicating an association between psoriasis, insulin resistance and type 2 diabetes [32, 33].

Apart from reasonable differences in ethnic origins and eating style, methodological differences might account for the apparent contrast in the association between carbohydrate intake and clinical severity of psoriasis reported in our study with that observed by Johnson et al. [11]. In particular, in our study, the nutritional status was evaluated using the 7-day food records, the effect of gender-specific food choice and the possible interference of the anti-psoriatic agents on metabolic profile were controlled by including only male treatment-naïve patients; furthermore, the diagnosis of psoriasis was clinically evaluated by PASI score and not self-reported.

A growing body of evidence indicates a common immune profile between psoriasis and MetS acting through increased Th type 1 proinflammatory cytokines [34]. Besides the well-known positive correlation between the clinical severity of psoriasis and MetS [1, 10], insulin resistance [32], and HS [2], we demonstrated also a novel association of PASI score with VAI and FLI, two surrogate indices of cardio-metabolic risk strictly correlated with MetS. This study demonstrates that the association between PASI and FLI is more significant than that with liver ultrasound. In particular, among HoMA-IR, VAI and FLI, the latter is the main predictor of the clinical severity of psoriasis. The novel association of the clinical severity of psoriasis with VAI and FLI supports the hypothesis of an early involvement in the inflammation milieu of psoriatic patients of other non-classical risk factors, i.e. HS and altered production of adipocytokines by dysfunctional adipocytes. According to these data, VAI and FLI can be suggested as early markers of the cardio-metabolic risk profile of psoriatic patients.

In our study, we found that the lowest intake of MUFA was the main predictor of the highest PASI score. The association between low MUFA consumption and progression of psoriasis is in line with the same observation reported in other chronic inflammatory diseases [16, 17]. MUFA are considered a healthy dietary fat, as opposed to SFA. The most frequently consumed MUFA rich dietary oils is EVOO. Traditionally, the beneficial effects of EVOO have been attributed to its high MUFA content (oleic acid) as it protects lipoproteins and cellular membranes from oxidative damage [35]. A low consumption of MUFA is a well-known mechanism contributing to the pathogenesis of Mets [36] and NAFLD in general population [37]. Besides their anti-oxidant and anti-inflammatory bioactive properties, MUFA may exert their beneficial effects on the pathogenesis of NAFLD through their influence on lipid metabolism either in the liver or the abdominal adipose tissue. In particular, a high-MUFA diet may avoid fat deposition in liver by the activation of catabolic pathways and could favor fatty acid deposition in adipose tissue enhancing the clearance of circulating triglyceride-rich lipoproteins by lipoprotein lipase [38, 39]. In this study, psoriatic patients presented lower MUFA consumption, worse lipid profile, and higher VAI score compared with their BMI-matched pairs. Moreover, the psoriatic patients with the lowest MUFA intake presented the highest values of PASI score and CRP levels. Of interest, in our group of psoriatic patients the association between psoriasis and metabolic syndrome appears to be independent of MUFA intake. In this respect, it is possible to speculate that the association of the low MUFA intake with the clinical severity of psoriasis might be based on the its adjunctive effect into increase the inflammation milieu of psoriatic patients, acting also independently of MetS, likely through the involvement of other non-classical risk factors, i.e. HS, expressed by FLI, and dysfunctional adipocytes, expressed by VAI.

We are aware that there are some limitations in the current study. First, the research suffers from a selection bias being limited to males subject only. This choice has been determined on the basis of the gender differences in the food pattern choices and in the anthropometric measures. Second, the sample size is small and a larger group of patients would have been more informative. However, in order to improve the power of the study, we increased the homogeneity of the studied group by including only treatment-naïve adult male patients, while treated patients were excluded to avoid the possible interference of the anti-psoriatic agents on metabolic profile. In addition, the diagnosis of psoriasis was not self-reported, but clinically evaluated using the PASI score. Finally, VAI and FLI are surrogate of adipocyte dysfunction and HS, respectively, but the reliability of FLI is not completely accepted by all researches. Nevertheless, the main strength of this study is the use of the 7-day food records. This method, which is considered as the “gold standard” in validation studies of different types of self-administered food frequency questionnaires, allows a more accurate measurement of the real dietary and macronutrient intakes compared to those obtained by retrospective food frequency questionnaires [40, 41].

## Conclusions

In conclusion, differences in macronutrient intake are present in adult male psoriatic patients compared to the control group. In the psoriatic patients, these differences are associated with the clinical severity of the disease and cardio-metabolic risk. Among cardio-metabolic indices, FLI, an accurate predictor of the HS, the hepatic manifestation of metabolic syndrome, might represent an early indicator of the cardio-metabolic risk profile of psoriatic patients. The low consumption of MUFA is the main predictor of the clinical severity of psoriasis and might act as a possible adjunctive mechanism in increasing the inflammation milieu of psoriatic patients, also independently of MetS. These results suggest a central role of Nutritionists in the management of psoriatic patients.

Future observational studies on larger population samples, comparing female and male psoriatic patients and long-term dietary intervention trials, will be critical for elucidate the global effects of macronutrient intake in psoriasis pathogenesis and progression.

## Abbreviations

BMI: Body Mass Index
PASI: Psoriasis Area and Severity Index
CRP: C-reactive protein
HoMA-IR: Homeostasis Model Assessment of Insulin Resistance
VAI: Visceral Adiposity Index
FLI: Fatty Liver Index
SFA: saturated fatty acids
MUFA: monounsaturated fatty acids
PUFA: polyunsaturated fatty acids
